# “Tip-in underwater endoscopic mucosal resection” without submucosal injection for superficial nonampullary duodenal adenomas

**DOI:** 10.1055/a-2134-9080

**Published:** 2023-08-21

**Authors:** Koichi Okamoto, Tomoyuki Kawaguchi, Kaizo Kagemoto, Yoshifumi Kida, Yasuhiro Mitsui, Yasushi Sato, Tetsuji Takayama

**Affiliations:** Department of Gastroenterology and Oncology, Institute of Biomedical Sciences, Tokushima University Graduate School, Japan

Underwater endoscopic mucosal resection (EMR) has been reported as an effective treatment for superficial nonampullary duodenal adenomas 1 2 . However, the en bloc and R0 resection rates have been relatively low 3 , possibly because the floating center of the lesion can hinder the visualization of its distal edge. This can cause the snare tip to slip, making it difficult to capture the lesion and achieve en bloc resection. To overcome these challenges, we report our new technique of “tip-in underwater EMR” for superficial nonampullary duodenal adenomas.

A 63-year-old-man presented with a duodenal adenoma with a white opaque substance that was 20 mm in diameter and located in the superior duodenal angulus ( Fig. 1 ). Saline was infused using a mechanical water pump (OFP-2; Olympus, Tokyo, Japan) ( Fig. 2 a ) to completely fill the lumen. The snare tip (Snaremaster; Olympus) was projected by 1–2 mm, and a mucosal incision was created on the distal edge of the lesion using a cutting current (Endo-cut I, Effect 2; VIO3, ERBE, Tubingen, Germany) ( Fig. 2 b ). The snare was then positioned around the lesion with gentle pushing ( Fig. 2 c ). After successful capture of the lesion, resection was performed using electrocautery (Endo-cut I, Effect 2). En bloc resection was achieved ( Fig. 2 d ) ( Video 1 ). Pathological findings indicated a low-grade adenoma with negative margins.

**Fig. 1:**
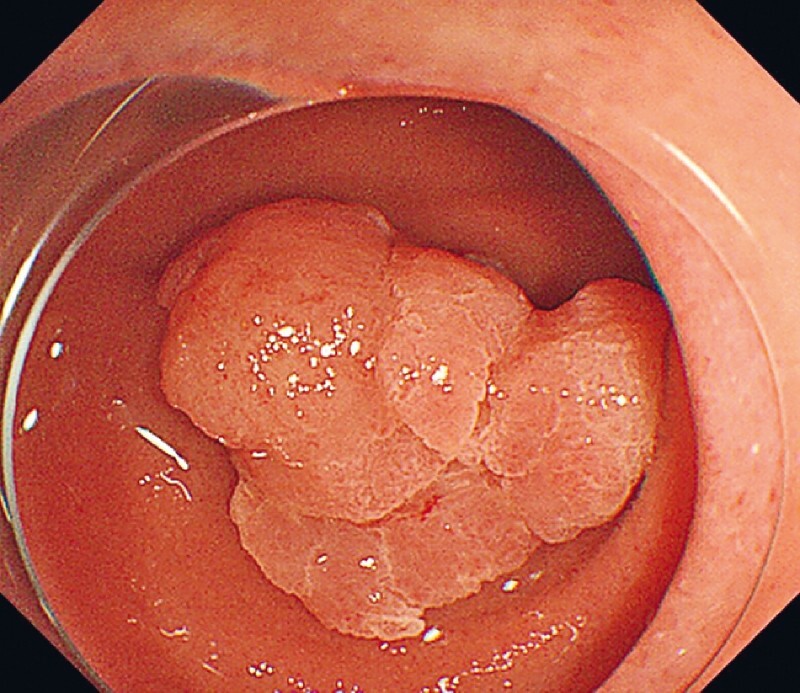
White light image showed a duodenal adenoma with white opaque substance.

**Fig. 2 a:**
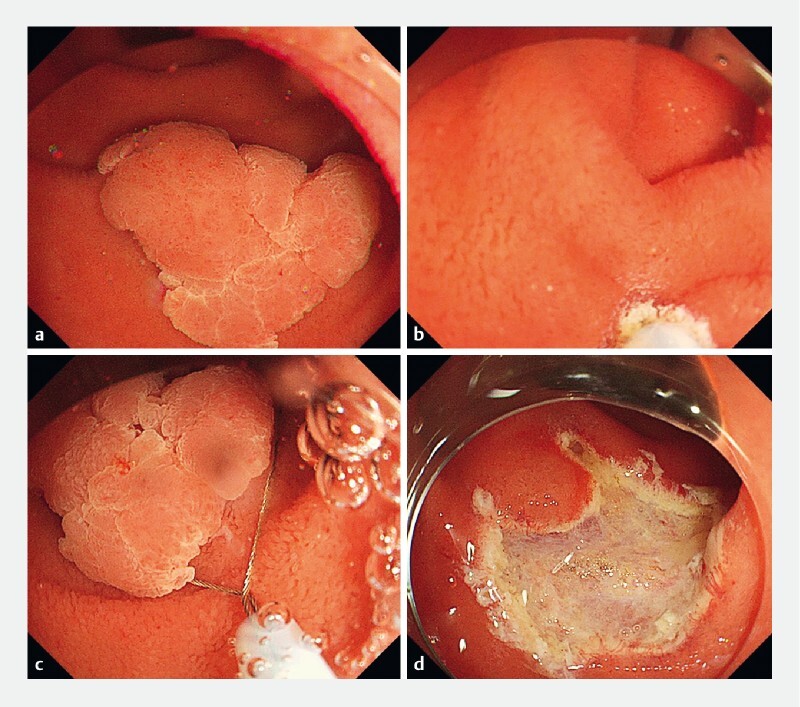
The lumen was filled with saline. **b** The distal edge of the lesion was cut using a cutting current without any submucosal injections. **c** The snare was positioned around the lesion. **d** There was no residual lesion after resection.

**Video 1** Tip-in underwater endoscopic mucosal resection for superficial nonampullary duodenal adenoma.


During tip-in EMR, making a pre-cut on the distal side of the lesion with prior submucosal injections and fixing the snare tip can make the snare less slippery 4 5 . However, our new technique of “tip-in underwater EMR” was performed without submucosal injections. Theoretically, the heat sink effect of water, combined with a relatively thicker wall, may protect against a transmural burn even while making a pre-cut with the snare tip. We are currently planning a feasibility study to demonstrate the utility and safety of this novel method.

Endoscopy_UCTN_Code_TTT_1AO_2AG

## Notes

Endoscopy E-Videos

https://eref.thieme.de/e-videos
*E-Videos* is an open access online section of the journal *Endoscopy* , reporting on interesting cases and new techniques in gastroenterological endoscopy. All papers include a high-quality video and are published with a Creative Commons CC-BY license. Endoscopy E-Videos qualify for HINARI discounts and waivers and eligibility is automatically checked during the submission process. We grant 100% waivers to articles whose corresponding authors are based in Group A countries and 50% waivers to those who are based in Group B countries as classified by Research4Life (see: https://www.research4life.org/access/eligibility/ ). This section has its own submission website at https://mc.manuscriptcentral.com/e-videos
